# Implementation and Outcomes of an Empiric Ivermectin *Strongyloides* Treatment Protocol for Patients Receiving High-Dose Corticosteroids for Severe COVID-19

**DOI:** 10.4269/ajtmh.23-0121

**Published:** 2023-07-17

**Authors:** Benjamin Swart, Aileen Ahiskali, Jack M. Wolf, Megan Shaughnessy

**Affiliations:** ^1^ Department of Internal Medicine, Hennepin Healthcare, Minneapolis, Minnesota;; ^2^ Department of Pharmacy, Hennepin Healthcare, Minneapolis, Minnesota;; ^3^ Division of Biostatistics, University of Minnesota, Minneapolis, Minnesota;; ^4^ Department of Infectious Disease, Hennepin Healthcare, Minneapolis, Minnesota

## Abstract

*Strongyloides stercoralis* is a parasitic roundworm that is present worldwide and can cause lifelong, often asymptomatic, infection. Immunosuppression, particularly by corticosteroids, is a risk factor for hyperinfection syndrome and disseminated strongyloidiasis—severe disease states that can lead to septic shock and death. Our institution implemented a strongyloidiasis screening and empiric ivermectin treatment protocol for inpatients receiving high-dose corticosteroids for severe COVID-19. Among 487 COVID-19 admissions treated with high-dose corticosteroids from June 10, 2020 to March 31, 2021, 61% of those with demographics at risk for *Strongyloides* exposure were screened for *Strongyloides* and treated empirically with ivermectin. Adherence to the protocol declined over time during the study period. The empiric ivermectin protocol appeared safe, but more research is needed to determine the effect on hyperinfection and/or disseminated strongyloidiasis risk and mortality rate, as well as to improve institutional adherence to the protocol.

## INTRODUCTION

*Strongyloides stercoralis* is a parasitic roundworm that causes the human disease strongyloidiasis, with a clinical syndrome ranging from asymptomatic infection to septic shock to death from disseminated disease (DD).^1^ It is found across multiple continents, with the greatest distribution in tropical regions, but is also found in temperate regions, particularly of low socioeconomic status.^2^ Patients can remain infected for life because of the parasite’s ability to complete its life cycle within a single human host by autoinfection. The preferred treatment of uncomplicated strongyloidiasis in immunocompetent adults is ivermectin 200 μg/kg daily for 2 days, although a recent study showed similar efficacy with a single dose.^1^^,^^3^ The former treatment regimen has a success rate of 98%, is relatively low cost and accessible, and is associated with minimal side effects.^4^ Treatment of strongyloidiasis in patients who are already, or soon to be, immunocompromised is less certain, and some experts recommend repeating the 2-day course of ivermectin approximately 2 weeks after administration of the initial treatment course.^5^

The most feared complications of strongyloidiasis are hyperinfection syndrome (HIS) and DD. In HIS, there is an increased parasite burden in expected regions of the body (skin, lungs, gastrointestinal tract). In DD, parasites migrate to any part of the body, potentially causing translocation of gastrointestinal bacteria into the bloodstream, leading to septic shock, with mortality rates ranging from 10% to 50%.^6^^,^^7^ Risk factors for HIS and DD include hematological malignancies, malnutrition, human T-cell lymphotropic virus type 1 infection, alcohol use disorder, and medication-induced immunosuppression.^1^ Glucocorticoids, including dexamethasone, are the most common immunosuppressive agents leading to HIS and DD, and previous reports have stressed the importance of screening for strongyloidiasis and/or empiric ivermectin treatment prior to corticosteroid induction.^8^ Screening for asymptomatic strongyloidiasis is typically done with commercially available serological tests, with sensitivities ranging from 70% to 95%, depending on the assay and immune status of the patient.^9^

In 2020, the rapid spread of the novel coronavirus, SARS-CoV-2, sparked the COVID-19 pandemic. Among many therapies trialed for treatment of COVID-19, glucocorticoids demonstrated efficacy in reducing mortality from severe disease.^10^ The COVID-19 pandemic also affected patients of lower socioeconomic status disproportionately, including immigrant populations.^11^ Hennepin County Medical Center (HCMC), part of the Hennepin Healthcare System, is a 484-bed safety net hospital in Minneapolis, MN, that serves high-risk patient groups, including a large immigrant and refugee population. Hennepin County Medical Center developed and implemented rapidly a systemwide protocol that defined dexamethasone eligibility, dosing, and duration for treatment of severe COVID-19. Given the rapid rise in dexamethasone use, HCMC also recognized that many patients receiving corticosteroids for severe COVID-19 were potentially at risk for HIS and DD. Scattered case reports have surfaced documenting cases of HIS and DD that occurred in the context of dexamethasone therapy for COVID-19.^12^^,^^13^ In response, the HCMC systemwide COVID-19 treatment protocol was updated to include strongyloidiasis screening and empiric ivermectin therapy for at-risk patients. Of note, use of ivermectin was never part of the treatment protocol for the primary treatment of COVID-19 because multiple studies have found it ineffective.^14^^,^^15^ Here we review the implementation of our strongyloidiasis screening and empiric ivermectin treatment protocol, as well as the characteristics and outcomes of HCMC patients with severe COVID-19.

## MATERIALS AND METHODS

The HCMC electronic medical record (EMR) was queried for all COVID-19 admissions from June 10, 2020 to March 31, 2021. Data collected included age, gender, hospital admission date, hospital discharge date, date of first positive COVID-19 test, dexamethasone (or equivalent) corticosteroid administration, date of *Strongyloides* IgG collection, *Strongyloides* IgG result, dates of ivermectin administration, receipt of other immunosuppressants, and patient-reported race, ethnicity, and primary language. Inclusion criteria included age 18 years or older, admission to the inpatient ward during the study period, a positive COVID-19 test within 30 days of index admission, and receipt of dexamethasone (or corticosteroid equivalent) per HCMC COVID-19 treatment protocol for 1 day or more. Exclusion criteria included readmission for COVID-19 if the patient had already received a corticosteroid during a previous admission, receipt of corticosteroid for an indication other than severe COVID-19, or revocation of permission to use patient information for research purposes.

Severe COVID-19 was defined as a positive polymerase chain reaction assay for SARS-CoV-2 and symptomatic infection requiring ≥ 2 L/min supplementary oxygen via nasal cannula, use of high-flow nasal cannula, or invasive/noninvasive mechanical ventilation. Patients meeting the criteria for severe COVID-19 but not requiring mechanical ventilation received 6 mg dexamethasone per ora (PO) daily for 10 days. Patients requiring mechanical ventilation received 20 mg intravenous (IV) dexamethasone PO for 5 days, followed by 10 mg IV dexamethasone PO for 5 days. If patients were from a tropical or semitropical area, loosely defined as anywhere the ground does not freeze, they were recommended to receive ivermectin empirically, 200 μg/kg daily, for 2 consecutive days and to undergo *Strongyloides* IgG serological testing. *Strongyloides* serology was an ELISA-based assay performed at Associated Clinical and University Pathologists, Inc., based in Salt Lake City, Utah. If the serology results were positive, it was recommended that patients received an additional 200 μg/kg ivermectin daily for 2 consecutive days approximately 2 weeks after their initial ivermectin course. *Strongyloides* serology results were available approximately 7 days after collection. If patients were from a region endemic for *Loa loa*, infectious diseases consultation was recommended prior to receiving ivermectin, given the risk of potentially fatal encephalopathy triggered by ivermectin use.^16^ Empiric ivermectin administration was started, ideally, prior to the first dose of corticosteroids; however, the protocol did not recommend delaying corticosteroid administration to give ivermectin.

Patients who received dexamethasone at doses outside of the institutional protocol or who received alternative corticosteroids (prednisone, methylprednisolone, etc.) were reviewed individually to confirm the steroid indication. If the indication was not for primary COVID (for example, chronic obstructive pulmonary disease exacerbation, but COVID positive without imaging characteristic of COVID), they were not included in our analysis.

As mentioned, patient demographics included patient-reported race, ethnicity, and primary language. Demographic categories were designed to be broadly representative of similar geography and climate for purposes of strongyloidiasis risk stratification and to fit more appropriately the categorical demographic information available in the EMR. Those who did not specify their race, ethnicity, or language were defined as “not specified.” Patients who reported their ethnicity/language as Russian, Caribbean Islander, or Puerto Rican, or whose self-reported race, ethnicity, and language aligned with multiple demographics, were defined as “other.”

A chart review was performed for every individual with positive *Strongyloides* serology and those who received additional doses of ivermectin outside of the empiric treatment protocol to determine whether there was suspicion for HIS or DD. We considered a suspicion for HIS or DD to be present if documented explicitly in care team or infectious diseases consultation notes, or if a sputum or stool ova and parasite examination was performed.

The total number of severe COVID-19 admissions receiving corticosteroids and the proportion of those admissions also administered ivermectin was calculated for each quarter (April–June 2020, July–September 2022, October–December 2022, and January–March 2021), both overall and within each demographic group. In-hospital mortality among those who received corticosteroids and who were *Strongyloides* serology positive was summarized overall and within each demographic group, conditional on whether ivermectin was prescribed. No formal statistical hypothesis testing was performed. All analyses were conducted using R version 4.04 (R Foundation for Statistical Computing, Vienna, Austria).

## RESULTS

From June 1, 2020 to March 31, 2021, there were 899 admissions for primary COVID-19 infection, representing 839 individual patients, of whom 487 (58%) received corticosteroids for severe COVID-19 pneumonia. Patient demographics (age, gender, and length of stay) are summarized within each demographic group in Table 1. Of those receiving corticosteroids, 153 of 487 (31%) received empiric ivermectin and 151 of 487 (31%) had *Strongyloides* serology screening. Patients with North American–Hispanic and South American demographics had the greatest rate of serology testing performed (70% and 74%, respectively). European and North American–Non-Hispanic demographics had the lowest rate of serology testing performed (< 10%). Eighteen of 151 *Strongyloides* serologies (12%) were positive, with the majority among the North American–Hispanic and sub-Saharan African demographics (Table 2).

**Table 1:** Demographics of COVID-19–positive admissions receiving corticosteroids

Demographic	*n*	Age, years; mean (SD)	Female, *n* (%)	Male, *n *(%)	Length of stay, days; mean (SD)
High-risk groups					
Central American	5	45 (9)	1 (20)	4 (80)	6 (6)
Middle Eastern and North African	4	54 (13)	1 (25)	3 (75)	6 (4)
North American–Hispanic	118	52 (14)	45 (38)	73 (62)	11 (13)
Southeast Asian and Indian	16	60 (17)	4 (25)	12 (75)	10 (9)
South American	19	56 (17)	3 (16)	16 (84)	9 (10)
Sub-Saharan African	49	62 (13)	20 (41)	29 (59)	18 (20)
Low-risk groups					
European	15	72 (13)	6 (40)	9 (60)	13 (16)
North American–Non-Hispanic	247	63 (14)	106 (43)	141 (57)	10 (12)
Other	7	73 (12)	3 (43)	4 (57)	6 (6)
Not Documented	7	67 (17)	1 (14)	6 (86)	13 (12)
Overall	487	60 (15)	190 (39)	297 (61)	11 (13)

**Table 2:** Characteristics of COVID-19–positive admissions receiving high-dose corticosteroids

Demographic	*n*	Received ivermectin, *n *(%)	*Strongyloides* serology collected, *n *(%)	*Strongyloides* serology positive, *n *(%)	*Strongyloides* serology collected and received ivermectin, *n *(%)
High-risk groups					
Central American	5	0 (0)	1 (20)	0 (0)	0 (0)
Middle Eastern and North African	4	2 (50)	1 (25)	0 (0)	1 (25)
North American–Hispanic	118	87 (74)	83 (70)	8 (6.8)	80 (68)
Southeast Asian and Indian	16	11 (69)	9 (56)	1 (6.2)	9 (56)
South American	19	15 (79)	14 (74)	2 (11)	13 (68)
Sub-Saharan African	49	31 (63)	29 (59)	6 (12)	26 (53)
Low-risk groups					
European	15	1 (6.7)	1 (6.7)	0 (0)	1 (6.7)
North American–Non-Hispanic	247	3 (1.2)	11 (4.5)	1 (0.4)	3 (1.2)
Other*	7	1 (14)	1 (14)	0 (0)	1 (14)
Not documented	7	2 (29)	1 (14)	0 (0)	1 (14)
Overall	487	153 (31)	151 (31)	18 (3.7)	135 (28)

* Other includes people from Russia, the Caribbean Islands, and Puerto Rico, and those whose self-reported race, ethnicity, and language aligned with multiple demographics.

Of those who received corticosteroids and had *Strongyloides* serology screening, 135 of 151 (90%) also received empiric ivermectin. Among the demographics at greatest risk for strongyloidiasis (North American–Hispanic, Southeast Asian and Indian, Sub-Saharan African, Middle Eastern and North African, Central and South American) who received corticosteroids, 129 of 211 (61%) had *Strongyloides* serology drawn and empiric ivermectin administered (Table 2). The three most common demographics at high risk for strongyloidiasis at HCMC were North American–Hispanic, Southeast Asian and Indian, and sub-Saharan African. More than 80% of those with North American–Hispanic and sub-Saharan African demographics who received corticosteroids and had *Strongyloides* serology screening also received ivermectin. Collectively, there were 318 people across all demographics who received high-dose dexamethasone without further *Strongyloides* testing or ivermectin administration. Eighty-two percent of these were in low-risk demographics and did not meet criteria for further interventions per our protocol. They were not monitored for other outcomes in our study.

**Figure 1.**
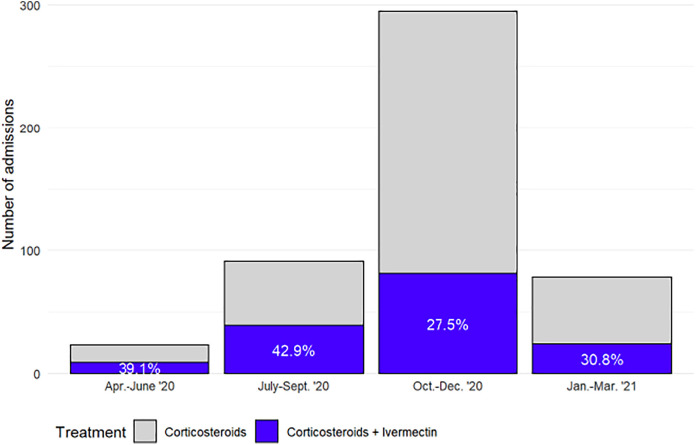
Proportion of severe COVID-19 admissions, by month, receiving corticosteroids and ivermectin.

During the study period, there was a decline in ivermectin administration among patients receiving corticosteroids (Figure 1). Approximately 40% of patients who received corticosteroids from April to September 2020 received ivermectin, whereas from October 2020 to March 2021, the number was nearer to 30%. The majority of COVID admissions occurred from October to December 2020, with approximately 28% of all COVID-positive patients admitted receiving ivermectin in this quarter.

**Figure 2.**
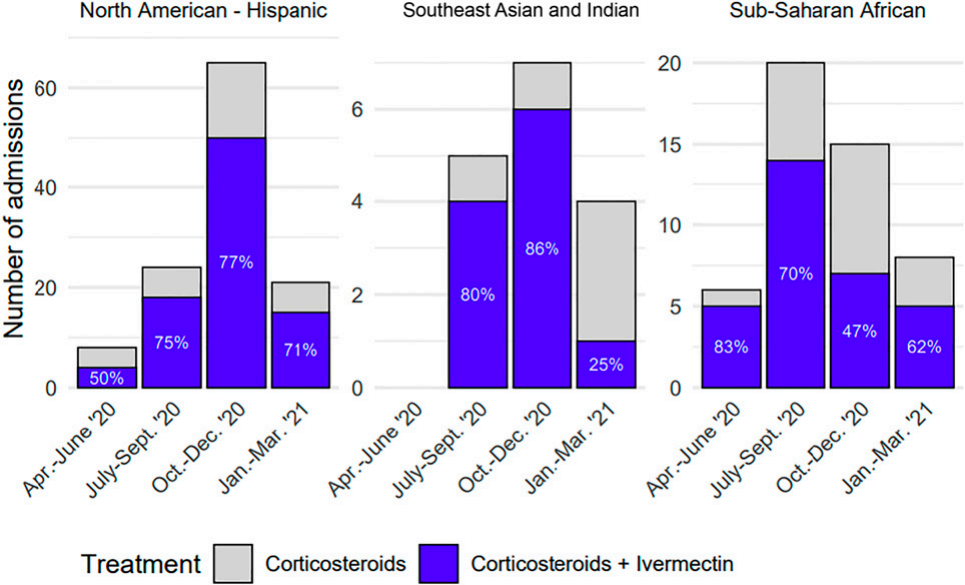
Proportion of patients, by month, admitted with severe COVID-19 receiving corticosteroids and ivermectin. The graphs correspond to the high-risk demographic groups present in the largest volume during the period studied.

Figure 2 shows the proportion of patients in each of the three main high-risk demographics receiving corticosteroids and ivermectin over the study period. Similarly, there is a trend toward lower rates of ivermectin administration later in the study period. Admissions for those in the sub-Saharan African demographic peaked slightly earlier than the Southeast Asian and North American–Hispanic demographics, and overall had a more consistent proportion of ivermectin administration during the study period.

Of those with positive *Strongyloides* serology, 15 of 18 (83%) received ivermectin (Table 3). Only one individual who screened positive for *Strongyloides* and did not receive ivermectin died during admission. This patient was not from a high-risk demographic, and it is unclear from our chart review why *Strongyloides* serology was performed. The chart review did not suggest HIS or DD as the cause of, or contributor to, death.

**Table 3:** Relationship between positive *Strongyloides* serology, receipt of ivermectin, and mortality

Demographic*	Received ivermectin		Did not receive ivermectin	
	Survived	Deceased	Survived	Deceased
North American–Non-Hispanic	0	0	0	1
North American–Hispanic	7	0	1	0
Sub-Saharan African	4	1	1	0
Southeast Asian and Indian	1	0	0	0
South American	2	0	0	0
Overall	14	1	2	1

* Middle Eastern and North African, European, Central American, Other, and Not Specified demographics were not included in this table because there were no positive *Strongyloides* serologies within these groups.

Among the patients included in our study and who were administered high-intensity corticosteroids, eight received tocilizumab. Five were in high-risk demographics. One patient had negative *Strongyloides* IgG and died of progressive hypoxemic respiratory failure, and another had positive *Strongyloides* IgG and was discharged to home after a largely uncomplicated hospital course. No patients who received tocilizumab had concerns for HIS or DD. Sarilumab was not used at our institution, and baricitinib was not implemented until after our trial period.

## DISCUSSION

High-risk groups for strongyloidiasis at HCMC were comprised primarily of the North American–Hispanic, Sub-Saharan African, and Southeast Asian and Indian demographics. Consistent with recommendations in the HCMC COVID-19 treatment guidelines, patients in these demographics also had the greatest rate of *Strongyloides* screening and empiric ivermectin administration. Concordantly, the North American–Non-Hispanic and European demographics had the least risk, and the lowest rate of *Strongyloides* screening and empiric ivermectin administration. Most conservatively, our protocol would have recommended screening and empiric ivermectin administration for all the North American–Hispanic, Middle Eastern and North African, Southeast Asian and Indian, and Central and South American patients, given their potential risk of strongyloidiasis. With the implementation of our protocol, 65% of those with high-risk demographics as defined earlier received *Strongyloides* screening and 62% received empiric ivermectin treatment.

Empiric treatment with ivermectin was limited by incomplete screening. When bundling screening with empiric treatment, 95% of individuals among high-risk demographics for *Strongyloides* infection who had serology collected also received ivermectin. It is unclear why some high-risk patients had screening performed but no empiric treatment. Of greatest concern are the three individuals who screened serology positive for *Strongyloides* but did not receive ivermectin treatment. On further chart review, one patient had received ivermectin 3 months prior to admission as part of a workup for kidney transplant, another was not given ivermectin because of a lack of oral access, and the third was unexplained. It is possible that simplifying the protocol to place more emphasis on empiric treatment with ivermectin, rather than screening, could have maximized appropriate ivermectin administration and better fit the goal of preventing HIS or DD in high-risk patients.

The rate of ivermectin administration declined during the study period, with the greatest treatment rates noted immediately after the empiric ivermectin guideline was added to the COVID-19 treatment protocol. Screening rates closely mirrored those of ivermectin administration. The exact etiology for this decline in screening and empiric ivermectin administration is unclear. One possibility is that providers in the hospital system became more confident in their knowledge of the COVID-19 treatment protocol as the rate of change in guidelines slowed, and therefore they were reviewing the treatment guidelines less frequently. This may have led to the decline in rates of supplemental testing such as *Strongyloides* screening when not referring to the guideline “checklist.”

Despite the lack of complete adherence to the empiric ivermectin guideline, there was only one case with suspicion for HIS or DD during the study period. The patient was a 30-year-old North American–Hispanic female with a past medical history of gestational diabetes mellitus and obesity. She was admitted in early 2021 for acute hypoxic respiratory failure requiring mechanical ventilation, with later evaluation confirming COVID-19. She was treated with dexamethasone and empiric ivermectin per guidelines upon admission. Her *Strongyloides* serology was found to be positive at 1.3 times the upper limit of normal. The infectious diseases team recommended bronchoscopy for sputum ova and parasite examination, which did not show evidence of *Strongyloides*. Her ivermectin course was repeated empirically 2 weeks after admission. She spent approximately 3 weeks in the medical intensive care unit and was ultimately discharged 4 weeks after admission to a long-term acute care hospital.

Of the three individuals who had positive *Strongyloides* serology and did not receive ivermectin, one died of progressive hypoxemic respiratory failure and acute blood loss anemia in the context of continuous renal replacement therapy while hospitalized in the intensive care unit. Another was discharged to home, and it is unclear, in retrospect, if this patient’s underlying hypoxemia was truly a result of COVID or secondary to another process with an incidental positive test. The third patient was discharged to home hospice care as a result of global deconditioning after a prolonged hospital stay, and died 1 month later with COVID symptoms largely resolved. None of these patients had a suspicion for HIS or DD as the main driver of their symptoms.

It is difficult to discern whether empiric treatment with ivermectin had a mortality benefit resulting from prevention of HIS and/or DD. There were few cases of suspected DD in our study, and none of the individuals with concern for DD died. Disparities in COVID-19 deaths in these demographic groups have been shown in many studies to have association with multiple confounding factors, including socioeconomic status, age, and medical comorbidities such as diabetes and cardiovascular disease.^11^^,^^17^ These variables were not taken into account because they were beyond the scope of our study.

One of the strengths of our study was the high number of immigrant patients available in our data set. Our inner-city safety net hospital cares for many foreign-born patients, and nearly half of the COVID-19 admissions included in our analysis were outside the North American–Non-Hispanic demographic. In addition, our health-care system is comprised of a single central hospital with multiple surrounding clinics, simplifying the standardization and implementation of protocols used in the inpatient setting. Within this single institution, there was streamlined coordination between infectious diseases and critical care specialists, specialized clinical pharmacists, and clinical researchers who were able to evaluate and implement up-to-date guidelines quickly to fit the local patient population and practice patterns more appropriately. In addition, all COVID-19 patients during the study period were admitted preferentially to specific inpatient teams, simplifying communication regarding COVID-19–related treatment guidelines and facilitating guideline adherence.

There are multiple limitations to our analysis, with the first being demographic categorization. Some patients may have been miscategorized by their language or ethnicity choice within our EMR. For example, a Somali-speaking American youth may have been born in Minnesota without subsequent international travel, but would have been categorized in the high-risk sub-Saharan African demographic in our analysis, although the actual risk of strongyloidiasis was low. In addition, the broad geographic groupings do not necessarily confer accurate strongyloidiasis risk profiles based on more detailed climate and socioeconomic status of the specific region within different countries. For example, there are large portions of Southeast Asia and South America where strongyloidiasis risk is low because of high elevations and cool temperatures, which prevent *Strongyloides* endemicity. However, patients from this area would still have been considered high risk. Temperate regions of the United States and Europe, particularly those areas with a lower socioeconomic status, are also endemic for strongyloidiasis; however, this level of specificity was not captured in our demographic data. Last, we were unable to assess for strongyloidiasis risk associated with travel to endemic areas because this information was not documented consistently or available in our EMR.

Our study is also limited by the lack of specificity in diagnosing chronic asymptomatic strongyloidiasis. We were unable to determine previously treated and resolved strongyloidiasis versus ongoing, active, chronic strongyloidiasis with serology screening alone. It is possible that some patients who screened positive did not have active infection at the time of their COVID-19 hospitalization and were not at risk for HIS or DD. This may be particularly true in Minnesota as a result of its relatively large refugee population. Since 1999, all US-bound refugees from regions endemic for strongyloidiasis (without concomitant *Loa loa* infection risk) receive empiric ivermectin treatment as part of predeparture presumptive antiparasitic therapy.^18^ However, other diagnostic modalities for strongyloidiasis—including stool ova and parasite, and peripheral eosinophilia—are fraught by lower sensitivity rates, and more intensive modalities such as culture and polymerase chain reaction are not widely available outside of research settings. There is also the possibility that patients with true chronic strongyloidiasis were missed in our study as a result of false-negative serology, which is more common among the immunocompromised or those receiving corticosteroids.^12^ A random review of cases does show that the majority of patients did have *Strongyloides* serology drawn at the time of initial steroid administration, which is likely reflective of COVID protocols implemented via EMR. This should have had minimal effect on outcomes, however, because the initial course of ivermectin should be given empirically regardless of serology results.

It is possible that some patients with potential HIS and/or DD were missed. Charts were not reviewed for patients who did not receive additional doses of ivermectin or who did not have *Strongyloides* serology collected during their hospitalization. There were many deaths that went uninvestigated among the nearly one third of individuals in high-risk demographics who received corticosteroids but were not screened or treated appropriately with ivermectin. This may have led to an underrepresentation of the true number of patients with possible HIS or DD. These complications in chronic strongyloidiasis are likely underdiagnosed in a variety of different disease conditions throughout the world, and we suspect the COVID-19 pandemic may be a prime example.

## CONCLUSION

In summary, implementation of an empiric ivermectin treatment guideline for patients hospitalized with severe COVID-19 at an urban safety net hospital serving a large immigrant population in the United States showed mixed success. Our guideline led to incomplete implementation of screening and empiric treatment of chronic strongyloidiasis, and adherence to the protocol decreased over time. This may indicate the need for simplification of guidelines and/or frequent reminders to providers caring for patients with severe COVID-19. This study was limited by difficulties in identifying, retrospectively, true strongyloidiasis risk, and highlights the importance of a thorough social history documenting a patient’s country of origin and travel history. Because of low rates of mortality in groups with positive *Strongyloides* serology and the difficulty in detecting cases of HIS or DD, further study is needed to determine whether an empiric ivermectin treatment protocol for strongyloidiasis has a morbidity or mortality benefit in this population.
